# *Nicotiana benthamiana* as a Transient Expression Host to Produce Auxin Analogs

**DOI:** 10.3389/fpls.2020.581675

**Published:** 2020-11-20

**Authors:** Katharine Davis, Danai S. Gkotsi, Duncan R. M. Smith, Rebecca J. M. Goss, Lorenzo Caputi, Sarah E. O’Connor

**Affiliations:** ^1^Department of Biochemistry, University of Cambridge, Cambridge, United Kingdom; ^2^School of Chemistry, University of St Andrews, St Andrews, United Kingdom; ^3^Department of Natural Product Biosynthesis, Max Planck Institute for Chemical Ecology, Jena, Germany

**Keywords:** indole-acetic acid, halogenase, combinatorial biosynthesis, auxin, unnatural natural product, new to nature products

## Abstract

Plant secondary metabolites have applications for the food, biofuel, and pharmaceutical industries. Recent advances in pathway elucidation and host expression systems now allow metabolic engineering of plant metabolic pathways to produce “new-to-nature” derivatives with novel biological activities, thereby amplifying the range of industrial uses for plant metabolites. Here we use a transient expression system in the model plant *Nicotiana benthamiana* to reconstitute the two-step plant-derived biosynthetic pathway for auxin (indole acetic acid) to achieve accumulation up to 500 ng/g fresh mass (FM). By expressing these plant-derived enzymes in combination with either bacterial halogenases and alternative substrates, we can produce both natural and new-to-nature halogenated auxin derivatives up to 990 ng/g FM. Proteins from the auxin synthesis pathway, tryptophan aminotransferases (TARs) and flavin-dependent monooxygenases (YUCs), could be transiently expressed in combination with four separate bacterial halogenases to generate halogenated auxin derivatives. Brominated auxin derivatives could also be observed after infiltration of the transfected *N. benthamiana* with potassium bromide and the halogenases. Finally, the production of additional auxin derivatives could also be achieved by co-infiltration of TAR and YUC genes with various tryptophan analogs. Given the emerging importance of transient expression in *N. benthamiana* for industrial scale protein and product expression, this work provides insight into the capacity of *N. benthamiana* to interface bacterial genes and synthetic substrates to produce novel halogenated metabolites.

## Introduction

Many natural products that share a common scaffold have subtle modifications to the core structure that dramatically modulate the biological function of the molecule (Pickens et al., 2011). The chemical similarity between product families mirrors a homology in genes featuring in their biosynthetic pathways. Notably, the encoded enzymes among very similar pathways can have altered substrate and product specificities. Mixing and matching enzymes from analogous pathways from different organisms, could enhance our chances of success in creating new-to-nature products with novel biological functions.

Halogenation is a particularly important chemical modification. In fact, the presence of a halogen affects multiple physiochemical properties of molecules including lipophilicity, size, polarity, and capacity for hydrogen bonding (Jeschke, 2010), which in turn affects biological activity. Notably, many pharmaceuticals and agrochemicals contain halogens. For example, the potent anti-cancer agent salinosporamide A (marizomib) uses a chlorine atom as a stable leaving group, a crucial feature for irreversible inhibition of the proteasome in cancerous cells (Miller et al., 2011).

**FIGURE 1:**
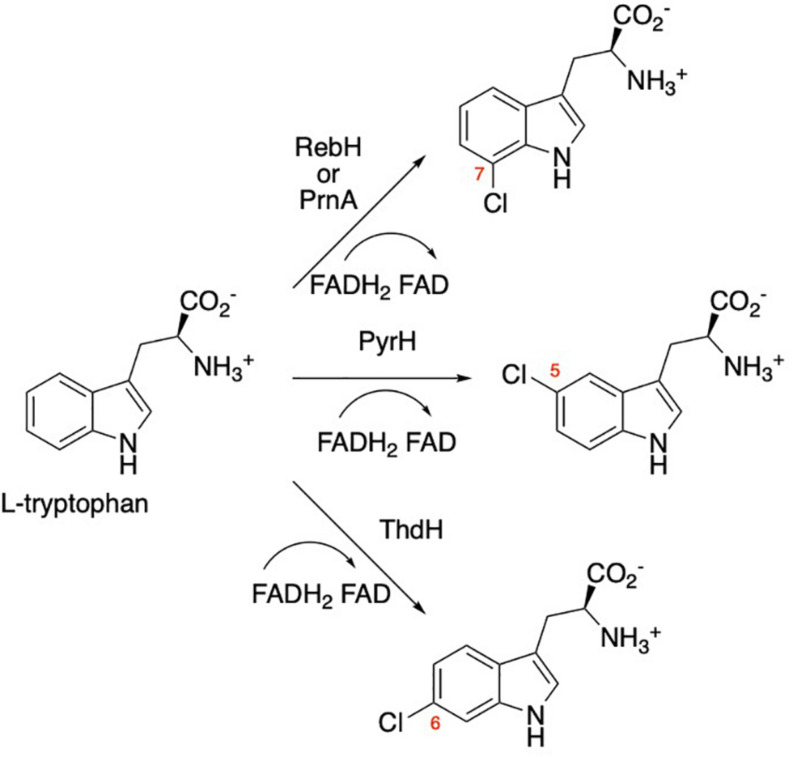
Regioselective halogenation of tryptophan by flavin-dependent bacterial halogenases RebH, PyrnA, PyrH, and ThdH. RebF is a flavin reductase (not shown) that recycles the flavin cofactor.

Halogenation in natural systems typically involves an enzymatic halogenation reaction (Neumann et al., 2008). For aromatic substrates, an FAD-dependent halogenase most commonly catalyzes the oxidation of a benign halide salt, readily available *in vivo*, to the corresponding hypohalous acid equivalent that is then installed in a regioselective manner on the aromatic substrate via an electrophilic aromatic substitution mechanism (Neumann et al., 2008; Figure 1). Among the most widely researched halogenases are the Trp-7 halogenases, PrnA (Keller et al., 2000) and RebH (Yeh et al., 2005), from *Pseudomonas fluorescens* and *Saccharothrix aerocolonigenes*, respectively, a Trp-6 halogenase, ThdH (Milbredt et al., 2014), from *Streptomyces albogriseolus* and a Trp-5 halogenase, PyrH (Zehner et al., 2005), from *Streptomyces rugosporus*. These enzymes have the unique ability to selectively halogenate the indole moiety of tryptophan under mild aqueous conditions. In contrast, synthetic halogenation of tryptophan and other aromatic compounds requires harsh reaction conditions and the reaction often lacks regioselectivity (Frese et al., 2014). Not surprisingly, there is substantial interest in using these flavin-dependent halogenases to replace or supplement current methods of chemically synthesizing halogenated compounds (Menon et al., 2016). Much effort has been made to expand the substrate scope and regioselectivity of these enzymes (Payne et al., 2013; Frese et al., 2014; Shepherd et al., 2016). Since these enzymes exhibit poor stability and low activity *in vitro* (Menon et al., 2016), *in vivo* systems are typically used for large-scale production of the halogenated metabolites. This approach has been used to produce antibiotics such as vancomycin (Jung et al., 2007) and griseofulvin (Saykhedkar and Singhal, 2004) in microbial host systems.

Although halogenation is widely observed among bacterial metabolites, this modification is relatively rare in terrestrial plants (Monde et al., 1998; Lam et al., 2015; Kim et al., 2020). However, there is an opportunity to use flavin-dependent halogenases from bacteria to create halogenated derivatives of plant natural products. For example, PyrH and RebH have been stably transformed into root cultures of the medicinal plant *Catharanthus roseus* to generate chlorinated tryptophan analogs that were subsequently incorporated into downstream alkaloid pathways (Runguphan et al., 2010). This approach relied on the native biosynthetic pathways of the host plant *C. roseus.* However, the model plant *Nicotiana benthamiana* is increasingly being used as factory in which secondary metabolite pathways are heterologously expressed and the resulting heterologous natural products can be produced in isolatable quantities (Reed and Osbourn, 2018). The method of agroinfiltration used to transiently transform the leaves of *N. benthamiana* allows the introduction of multiple enzymes by simply mixing together *Agrobacterium tumefaciens* strains harboring different biosynthetic genes (Sainsbury and Lomonossoff, 2014). This means that entire biosynthetic pathways, and combinations thereof, can be readily transformed into and expressed by this plant. Since recent work has demonstrated that bacterial halogenases can be functionally expressed in *A. thaliana* (Walter et al., 2020), *Brassica rapa* (Neumann et al., 2020), and *N. benthamiana* (Frabel et al., 2016), we set out to use *N. benthamiana* as a host for incorporation of bacterial halogenases into heterologous plant pathways to generate novel halogenated products.

**FIGURE 2:**
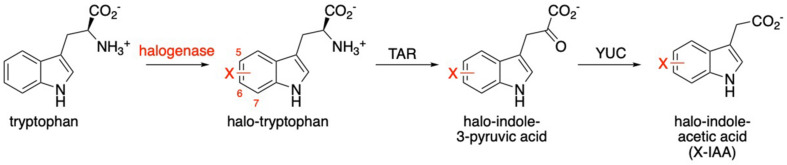
Production of indole-3-acetic acid (IAA) from tryptophan. The role of the bacterial halogenase (incorporating halogen X, typically Cl) is indicated in red.

Although previous work in our group established that halogenated monoterpene indole alkaloids can be produced in *C. roseus* root culture that is transformed with a bacterial halogenase (Runguphan et al., 2010), heterologous reconstitution of monoterpene indole alkaloid pathways (>10 enzymes) presents a formidable metabolic engineering challenge that is still being addressed. Instead, we chose to use a shorter plant-derived pathway as a test case for this study. Indole-3-Acetic Acid (IAA) is the most common auxin in higher plants, with a key role in plant growth and development (Cook and Ross, 2016). IAA can be formed from hydrolytic cleavage of IAA conjugates such as sugars, amino acids and peptides (Yang et al., 2008), but it is also produced directly via a *de novo* synthesis pathway (Mano and Nemoto, 2012). The synthesis of terpene indole alkaloids and the synthesis of IAA have a key similarity in that they are derived from tryptophan (O’Connor and Maresh, 2006). Here the similarity ends as the major IAA production pathway involves first a member of an aminotransferase family (TAR) that converts tryptophan to indole pyruvic acid (Tao et al., 2008; Zheng et al., 2013). This intermediate undergoes oxidative decarboxylation by a flavin-dependent monooxygenase (YUC) in the second step to produce IAA (Gao et al., 2016; Figure 2). Synthetic auxin analogs have been produced through chemical synthesis (Caboche et al., 1987), and a number of these derivatives display altered bioactivities. One halogenated auxin, 4-Cl-IAA, is produced naturally in certain members of the Fabaceae plant family, through the conversion of 4-Cl-Trp to Cl-IPA and finally to Cl-IAA (Tivendale et al., 2012), though the source of this halogenated tryptophan is still unknown (Karcz and Burdach, 2002). The accumulation of 4-Cl-IAA during the seed development in *Pisum sativum*, suggests that the enzymes involved in its biosynthesis are able to handle halogenated substrates. Therefore, we looked at this plant as a source of enzymes for our study.

The short, modular pathway of IAA seemed to be well-suited for combinatorial biosynthesis in *N. benthamiana* using a deconstructed plant virus, an approach in which genes from various biosynthetic pathways are mixed and matched to create new-to-nature products with potentially novel biological functions (Pickens et al., 2011). Here we express the 2-step IAA pathway using *TAR* genes from *Arabidopsis thaliana* and *YUC* genes from *P. sativum.* An agroinfiltration approach was applied to allow transient expression of multiple genes, including that of multiple bacterial halogenases, to produce novel halogenated IAA analogs *in vivo*. Additional auxin derivatives were produced by co-infiltration of the biosynthetic genes with various tryptophan analogs. These experiments demonstrate the capacity of *N. benthamiana* to produce a suite of halogenated products using combinatorial biosynthesis.

## Results

### Heterologous Expression of TAA and YUC to Produce IAA in *N. benthamiana*

We first assembled the suite of genes required for IAA biosynthesis. The members of the TAR aminotransferase family in *A. thaliana* are named *TAA1* (AT1g70560) and *TAR1-4* (AT1G23320, AT4G24670, AT1G34040, AT1G34060). These *A. thaliana* genes were selected for this study since their biochemical activity has been examined previously (Stepanova et al., 2008). The genes *PsTAR1* (JQ002582.1) and *PsTAR2* (JQ002584.1) have also been characterized (Caboche et al., 1987). Our preliminary experiments did not reveal any substantial difference in activity between the various aminotransferases when expressed in *N. benthamiana* but suggested that co-expression of two enzymes together slightly improved the conversion (data not shown). Therefore, all our experiments were performed using both *AtTAA1* and *AtTAR2*. The second and last step of IAA biosynthesis is catalyzed by the flavin containing YUC enzymes. Whilst the *YUC* gene family in *A. thaliana* is known and some of the genes have been extensively characterized (Kim et al., 2007; Dai et al., 2013), information about the YUC genes in *P. sativum* is limited. In fact, only two genes, named *PsYUC1* (HQ439907.2) and *PsYUC2* (HQ439908.1) have been identified but not fully characterized at a functional level (Tivendale et al., 2010). Therefore, we performed RNA sequencing of developing pea seeds to identify all of the YUC genes expressed during the accumulation of 4-Cl-IAA. Eight genes were identified (PsYUC1-8, accession codes can be found in Supplementary Table S1) and were cloned from a cDNA library of the same tissues.

**FIGURE 3:**
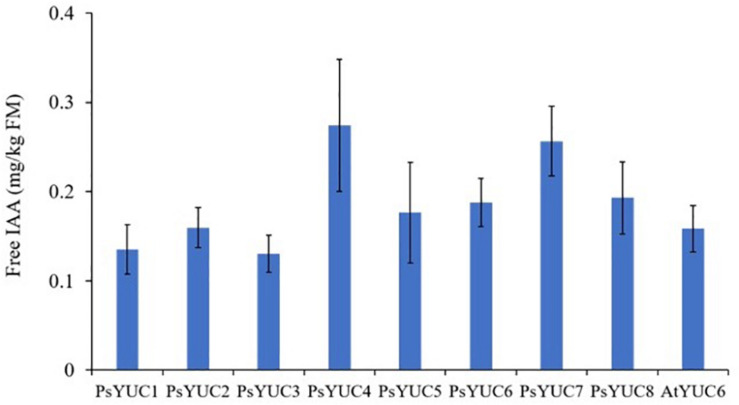
Free IAA production by *N. benthamiana* leaves above background levels following co-infiltration of AtTAA1, AtTAR2 and one of nine YUC genes. In each case, bars show the mean ± SEM, *n* = 4. The accumulation of free IAA in control plants infiltrated only with empty vector was below quantifiable levels so it is not shown in the figure.

Following the expression of *AtTAA1* and *AtTAR2* and *YUC* genes (*PsYUC1-8* or *AtYUC6*) in *N. benthamiana* leaves, free IAA accumulation was observed by UPLC/MS in transformed leaf tissue extracts (Figure 3). The production of free IAA from control plants infiltrated only with empty vector was below quantifiable levels. A total of 9 *YUC* genes from *A. thaliana* and *P. sativum* were tested with both *AtTAA1* and *AtTAR2* and although expression of each construct combination led to IAA production, the highest yields (approximately 500 ng/g FM) were observed with *PsYUC7* and *PsYUC4*. The identity of the product was confirmed using UPLC-MS by comparison with an IAA standard (Supplementary Figure S1) and the quantification was based on a calibration curve. As there was no significant difference between the yield of IAA from expression of *PsYUC4* and *PsYUC7*, we arbitrarily chose *PsYUC7* to perform all further experiments requiring the YUC monooxygenase. Plants expressing the combination of *TAR* and *YUC* genes displayed a curly leaf phenotype, consistent with auxin over-production (Klee et al., 1987; Jackson et al., 2002; Supplementary Figure S2).

### Production of Chlorinated Tryptophan and Chlorinated IAA in *N. benthamiana*

**FIGURE 4:**
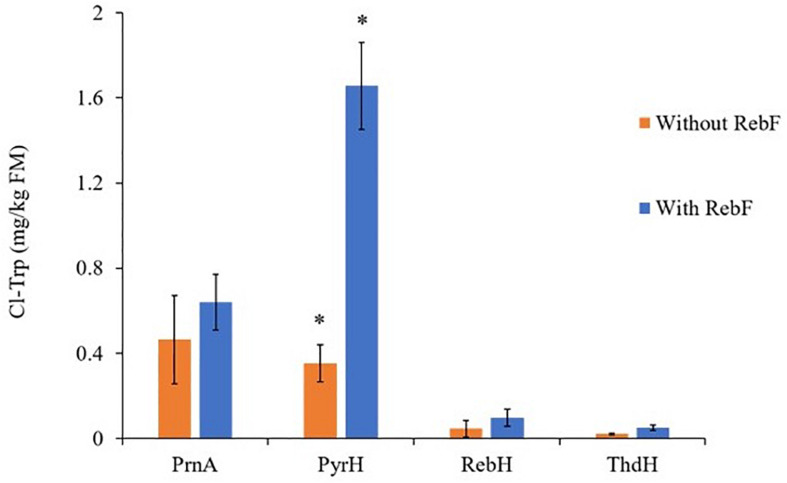
Cl-Trp production above background levels by *N. benthamiana* leaves following infiltration of a flavin-dependent halogenase with and without the partner reductase, RebF. In all cases bars show mean ± SEM, *n* = 4. * indicates significant difference following analysis by a two-sample, two-tailed Welch’s *t*-test in RStudio, *p* ≤ 0.005. A Shapiro–Wilks test was used to verify that the Cl-Trp production by each replicate leaf followed a normal distribution. The outcome of the *t*-test cannot be relied upon for the comparison between RebH +/– RebF, as the Cl-Trp replicate yields for RebH – RebF are not normally distributed.

Expression of each of the bacterial halogenases, *RebH*, *PyrH*, *PrynA* and *ThdH*, in *N. benthamiana* led to production of chlorinated tryptophan (Cl-Trp) (Figure 4). It has been reported previously that when expressed in cytosol *in planta*, *RebH* was capable of producing only trace amounts of halogenated tryptophan without the presence of the partner flavin reductase, *RebF* (Frabel et al., 2016). In the present study, each halogenase was expressed both with and without the FAD-reductase *RebF*, and in each case Cl-Trp could be observed by UPLC/MS (Supplementary Figure S3). The co-expression of *RebF* improved the yield of Cl-Trp only in the case of *PyrH*, whilst for the other halogenases no significant differences were observed. Identification of the chlorinated tryptophan products was performed by comparison using UPLC-MS with authentic 5-, 6-, and 7-chlorotryptophan standards (Supplementary Figure S4).

**FIGURE 5:**
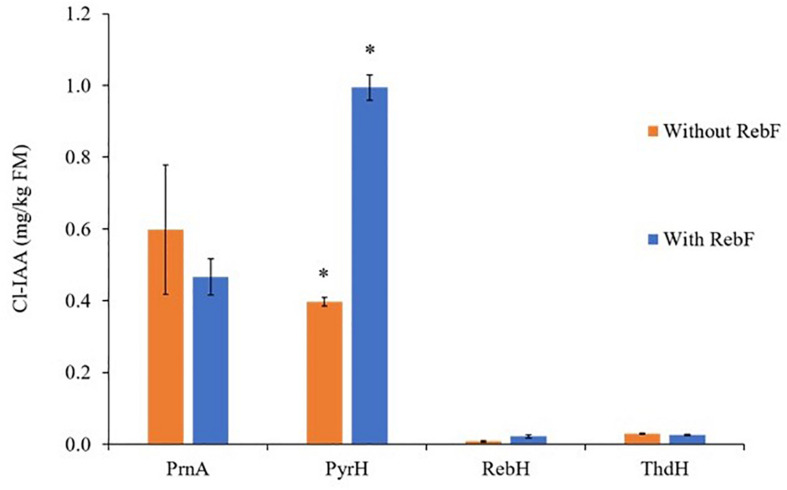
Free Cl-IAA production above background levels in *N. benthamiana* leaves following co-infiltration of AtTAA1, AtTAR2, PsYUC7 and one of four flavin-dependent halogenases with and without the partner reductase, RebF. Bars represent mean ± SEM, *n* = 4, except for RebH without RebF and PyrH with RebF for which *n* = 3. * indicates significant difference following a two-sample, two-tailed Welch’s *t*-test in RStudio, *p* ≤ 0.005. A Shapiro–Wilks test was used to verify that the Cl-Trp production by each replicate leaf followed a normal distribution. The outcome of the *t*-test cannot be relied upon for the comparison between ThdH+/– RebF, as the replicate Cl-IAA yields for ThdH + RebF are not normally distributed.

Expression of each of the bacterial halogenases individually with *AtTAA1*, *AtTAR2*, and *PsYUC7* led to production of Cl-IAA derivatives with the predicted regioselectivity (Figure 5). As above, the halogenases were active both in the presence and absence of *RebF*, and in both cases Cl-IAA was observed. The presence of *RebF* made a significant difference to the yield of Cl-IAA in the case of *PyrH*, leading to the accumulation of up to ∼990 ng/g FM of Cl-IAA. The identity of Cl-IAA was confirmed by comparison with peak retention times of relevant authentic 5-, 6-, and 7-Cl-IAA standards (Supplementary Figure S4). Beyond the curling leaf phenotype characteristic of auxin over-production, no additional phenotype was observed in plants producing halogenated versions of tryptophan or IAA.

### Production of Brominated IAA in *N. benthamiana*

RebH has been reported to install bromine onto the 7-position of tryptophan *in planta* following transformation in buffer containing KBr (Frabel et al., 2016). By infiltrating *AtTAA1, AtTAR2*, *PsYUC7, RebF* and each of the bacterial halogenases in buffer containing KBr, the production of Br-Trp and Br-IAA was achieved (Table 1). Although the production of Br-Trp was observed with all the halogenases tested, only transformations with *PrnA*, *PyrH*, and *RebH* genes led to successful production of Br-IAA (Supplementary Figure S5). In particular, of the total halogenated IAA *PrnA* produced 9.6% of Br-IAA, *PyrH* 3.5%, and *RebH* 17.6%. The identity of brominated tryptophan was confirmed using UPLC-MS, by comparison with authentic Br-Trp standards (Supplementary Figure S6).

**TABLE 1:** Halo-IAA production results following halogenase infiltration.

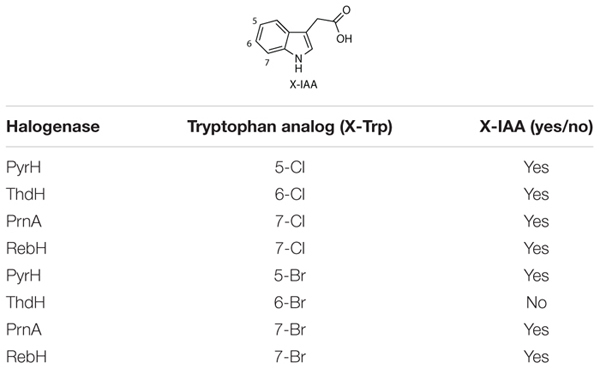		

*Yes/no indicates whether, following infiltration of AtTARs, PsYUC7 and one of four bacterial halogenases, halo-IAA could be produced in *N. benthamiana* leaves. In each case, the tryptophan analog could be observed.*

### Infiltration of Tryptophan Analogs for Additional IAA Analogs

Commercially available tryptophan analogs that cannot be readily accessed by known halogenases (Table 2) were infiltrated in *N. benthamiana* leaves, along with *AtTAA1, AtTAR2*, and *PsYUC7*. Formation of the corresponding IAA analogs was then assessed by LC-MS based metabolite analysis of the infiltrated leaves (Supplementary Figure S7). Although successful conversion to a product with a mass corresponding to the expected IAA derivative was observed in some cases, no IAA formation was observed for the fluoro-tryptophan series, for 7-Br-Trp and for 5-OH-Trp. We speculated that the electronic properties of the fluoro series and the 5-OH-Trp could interfere with enzyme recognition. We attributed the failure to produce 7-Br-Trp after infiltration to the poor solubility of this substrate in the infiltration buffer, which thus was not available to the enzymes. In fact, 7-Br-Trp also accumulated upon co-infiltration of KBr with the halogenases. Due to the lack of standards for the IAA analogs, the yields of these novel metabolites could not be quantified accurately.

**TABLE 2:** Results from Trp-analog infiltration experimentation.

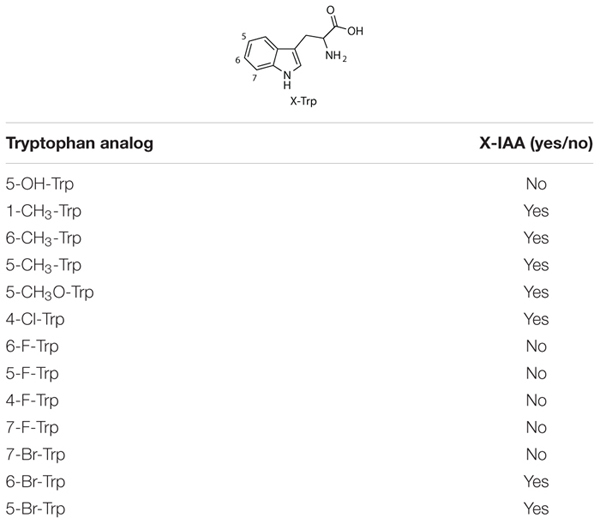	

*Yes/No indicates whether, following infiltration of the Trp-analog, *N. benthamiana* plants co-infiltrated with AtTARs and PsYUC7, produced a compound with a mass matching the corresponding IAA analog in observable levels, in at least two of three biological repeats, following metabolite extraction and analysis by LCMS.*

## Discussion

In this study, we explored the use of combinatorial biosynthesis to produce novel analogs of IAA in *N. benthamiana*, with a particular focus on generating halogenated analogs. Agroinfiltration was used to transiently express genes from the IAA pathway in *N. benthamiana* leaves. The background level of free IAA in the *N. benthamiana* leaf was below the limit of detection of our UPLC-MS analysis. While the specific pathway for auxin production in *N. benthamiana* is not currently known, the lack of free IAA was likely due to conversion of native IAA to conjugates to both spatially and temporally regulate the concentrations of this growth hormone (Walter et al., 2020). Over-production of auxin was also evident in transformed leaves by an apparent downward-curled phenotype. Previous literature has described this phenotype as being due to non-homeostatic levels of IAA affecting the regulation of adaxial (upper leaf layer) and abaxial (lower leaf layer) cell growth (Klee et al., 1987; Jackson et al., 2002).

To produce chlorinated IAA, chlorinated tryptophan substrate was first produced by expressing one of four bacterial halogenases individually in *N. benthamiana*. Relying on the cytosolic supply of tryptophan, the highest amount of Cl-Trp product (5-chlorotryptophan) was achieved using *PyrH* (∼1.66 μg/g FM). All four bacterial halogenases have similar K_*m*_ and k_*cat*_ values for chlorination of tryptophan (Pée et al., 2016), so the observed difference in product yield between the halogenases may be due to differences in transcription, translation or other factors. However, considering that all enzymes were produced in the plant using the same virus-derived vector that mediates episomal expression, we could hypothesize that the different product yields could results from different protein stabilities. *PyrH* was the only halogenase that showed a significantly higher production of Cl-Trp in presence of a partner reductase (*RebF*). In absence of *RebF*, the endogenous supply of FADH_2_ may have been sufficient for production of Cl-Trp by each halogenase (Figure 4). However, given the higher titers of chlorotryptophan that were obtained using *PyrH*, the endogenous supply of FADH_2_ may have been a rate-limiting factor for this halogenase. A previous study has shown that cytosolic expression of *RebH* in *N. benthamiana* leaves, in the absence of *RebF*, produces only trace amounts of Cl-Trp (Frabel et al., 2016). Only when localized to the chloroplast was a partner reductase unnecessary to produce quantifiable levels of Cl-Trp (Frabel et al., 2016). Although the data in our investigation are not entirely consistent with this, as quantifiable levels of Cl-Trp were achieved by cytosolic *RebH* in absence of *RebF*, it should be noted that the yield of Cl-Trp achieved is lower than that achieved by Frabel et al. (2016) in the chloroplast: up to ∼77.8 ng/mg, in comparison to ∼0.1 ± ng/mg achieved in this investigation. This difference could be due to higher levels of FADH_2_ and tryptophan in the chloroplast than in the cytosol.

Previous studies have confirmed the ability of bacterial halogenases to incorporate bromine as well as chlorine into a tryptophan scaffold *in vitro*. *In planta*, Frabel et al. (2016) achieved production of brominated tryptophan by infiltrating *N. benthamiana* with *A. tumefaciens* expressing *RebH* and *Stth* (coding for a tryptophan-6 halogenase) in buffer containing 40 mM KBr. Similarly, following infiltration of *PyrH*, *RebH*, and *PrnA* with *AtTARs* and *PsYUC7* in KBr buffer, a compound with a mass corresponding to Br-IAA was observed in this investigation. Cl-IAA was also produced following infiltration with buffer containing KBr (Supplementary Figure S6), suggesting that the bacterial halogenases readily utilize the *in-planta* supply of chloride for tryptophan chlorination. By infiltrating *N. benthamiana* with tryptophan analogs, rather than bacterial halogenases, additional novel IAA analogs were produced, though the lack of appropriate standards prevented quantification and rigorous structural characterization of these novel products.

In this proof-of-concept study, we demonstrate that both directed biosynthesis and combinatorial biosynthesis can be used to generate halogenated metabolite analogs in a transient *N. benthamiana* expression system. Plants are increasingly being used as effective natural product factories due to their low-maintenance growth requirements (light, water, and nutrients) and also to the development of highly efficient transient expression systems. This study highlights how *N. benthamiana* can serve as a robust platform to rapidly access a library of halogenated derivatives through the incorporation of FAD-dependent halogenases into an established plant biosynthetic pathway. The new-to-nature auxin analogs generated within this investigation may therefore have applications in the study of plant developmental biology but more importantly, the possibility to produce tryptophan analogs *in planta* will provide access to precursors for further biosynthetic steps leading to compounds with diverse biological activities, such as molecules with anticancer, antiarrhythmic, and antidiabetic activities. This work provides a platform for tryptophan-based combinatorial biosynthesis, and is a starting point for optimization of gene expression and downstream processing to increase productivity and yields of the final products.

## Materials and Methods

### *P. sativum* Developing Seeds RNA Sequencing

*Pisum sativum* seeds (cultivar Cameor) were obtained from the Germplasm Resources Unit at the John Innes Centre United Kingdom and were grown in a glasshouse at 24/16°C day/night temperature with a 14-h photoperiod and at 70% relative humidity. The developing seeds were collected between 7 and 21 days after floral anthesis and the RNA was extracted using a RNeasy Plant mini kit (Qiagen). RNA sequencing was performed at the Earlham Institute (United Kingdom). Briefly, Illumina barcoded TruSeq RNA library were prepared and sequenced using Illumina HiSeq 2000/2500 in High-Output mode using 100 bp paired-end reads to generate at least 100 million pairs of reads per lane. FASTQC^1^ was used on the reads to assess the quality. Trinity software (Grabherr et al., 2011) was used to assembly a *de novo* transcriptome from the paired-end reads with the default parameters after removal of low-quality reads and trimming. *PsYUC* genes were identified by blasting the *AtYUC6* sequence into the assembled transcriptome and retrieving the closest homologs.

### Gene Cloning and *Agrobacterium tumefaciens* Transformation

Genes in Supplementary Table S1 were amplified by PCR from a cDNA library prepared using the SuperScript III kit (Invitrogen) using primers with vector specific over-hangs (Supplementary Table S2) and a high-fidelity polymerase (CloneAmp HiFi PCR Premix, Clontech-Takara). The PCR product was gel-purified and ligated using In-Fusion HD Cloning Kit (Clontech-Takara) into a modified TRBO (Lindbo, 2007) vector, in which the cloning cassette of the pOPINF (Berrow et al., 2007) vector was inserted in the *NotI* restriction site. Codon optimized bacterial halogenase genes *RebH*, *PyrH*, *PrynA* and *ThdH* and the flavin reductase *RebF* with the cloning overhangs were ligated into the linearized vector using In-Fusion HD Cloning Kit (Clontech-Takara). Competent Stellar *E. coli* cells were transformed with the constructs and selected on Luria-Bertani (LB) agar plates containing 50 μg/ml kanamycin. Colony PCR and Sanger sequencing were used to confirm the correct insertion of the genes. Electroporation was used to transform 50 μL aliquots of competent *Agrobacterium tumefaciens* (strain GV3101) cells with 2 μL of each plasmid (100–200 ng of DNA) containing a gene of interest. Following recovery in non-selective SOC medium for 2 h at 28°C, cells were plated on selective LB-agar containing 50 μg/mL kanamycin, 50 μg/mL rifampicin, and 25 μg/mL gentamicin and grown for 48 h at 28°C. Colonies formed were used to inoculate 10 mL aliquots of selective LB media in universal tubes and grown for 48 h at 28°C and 200 rpm. The cells were collected by centrifugation at 4000 × *g* for 10 min, resuspended in 10 mL infiltration buffer (10 mM NaCl, 1.75 mM CaCl_2_, 100 μM acetosyringone) to an OD_600_ of 1.0 and incubated at room temperature for 2 h.

### *N. benthamiana* Transformation and Harvesting

*Agrobacterium tumefaciens* suspensions were diluted to an OD_600_ of 0.1 or 0.5 in infiltration buffer, as the OD_600nm_ of the injected *A. tumefaciens* culture does not seem to impact significantly the amount of protein produced (Buyel and Fischer, 2012). Combinations of constructs were made to infiltrate each leaf with genes for completing multiple steps of the biosynthetic pathway. Control plants were infiltrated with cultures containing the modified TRBO vector, without an insert. Four leaves of 3-week-old *N. benthamiana* plants, grown in climate chambers at 24/21°C day/night temperature with a 16-h photoperiod and at 70% relative humidity, were infiltrated by injection with each bacterial suspension, forming four replicates for each construct combination. Syringes (1 mL, without needle) loaded with each bacterial culture were used to infiltrate the entire leaf from the abaxial side. Exogenous tryptophan substrates, if used, were infiltrated by the same method on the 4th day of growth as 100 μM solutions in infiltration buffer. Plants were harvested 5 days following infiltration with bacterial culture; harvested leaves were frozen in liquid nitrogen, individually ground to a powder using a pestle and mortar and stored at −80°C until extraction.

For Br incorporation the above protocol was used, replacing NaCl buffer with KBr buffer (40 mM KBr, 100 μM acetosyringone).

### Metabolite Extraction

Metabolite extraction was performed using 200 mg of each powdered leaf sample, following a method described previously (Novák et al., 2012). Briefly, samples were vortexed in 1 mL phosphate buffer (100 mM K_2_HPO_4_, pH 7.0) and sonicated in a water bath for 5 min. Samples were centrifuged for 10 min at top speed in a table-top centrifuge, 7 μL 1 M HCl were added to the supernatants (pH to c.a. 2.7). Samples were centrifuged for a second time for 5 min. Supernatants were passed through an Oasis HLB solid phase extraction (SPE) column, washed with 1 mL of 5% MeOH and metabolites were eluted with 200 μL of 80% MeOH. This method was used for all harvested plant tissue except for the infiltrated 5-OH-Trp sample, for which the SPE column was washed with phosphate buffer prior to elution in 80% MeOH.

### Metabolite Analysis by LC/MS

Ultra-performance liquid chromatography-Tandem mass spectrometry (UPLC-MS/MS) analysis of the metabolites was carried out on a Waters Acquity UPLC system connected to a Xevo TQS (Waters) spectrometer. Chromatography was performed using an Acquity BEH C18 1.7 μm 2.1 × 50 mm column kept at 35°C. Water containing 0.1% formic acid and acetonitrile containing 0.1% formic acid were used as mobile phases A and B, respectively with a flow rate of 0.35 mL min^–1^. The gradient was 1% B from 0 to 6 min followed by a linear gradient to 60% B for 6 to 6.5 min followed by isocratic 60% B. The injection volume of both the standard solutions and the samples was 2 μL. Samples were kept at 10°C during the analysis. MS detection was performed in positive ESI. Capillary voltage was 3.0 kV; the source was kept at 150°C; desolvation temperature was 500°C; cone gas flow, 100 L h^–1^; and desolvation gas flow, 800 L h^–1^. Unit resolution was applied to each quadrupole. Multiple reaction monitoring (MRM) signals, optimized using authentic standards, were used for detection and quantification of chlorinated tryptophan. MRM signals for IAA derivatives generated in this study from tryptophan analogs, for which we did not have standards, were predicted based on the fragmentation patterns and collision energies of halogenated IAA (Supplementary Table S3).

### One-Pot Biotransformation of L-Halotryptophans With Tryptophan Synthase

The indole substrate (2 mmol) and serine (1.25 eq, 2.5 mmol) were suspended in 100 ml of reaction buffer (100 mM potassium phosphate, adjusted to pH 7.8). The crude lysate containing the overexpressed tryptophan synthase enzyme from *Salmonella enterica* [protocol for production detailed in Smith et al. (2014)] was thawed in a 37°C water bath, and 3 ml of lysate contained in cellulose tubing were used for each biotransformation. The biotransformations were incubated at 37°C with shaking at 180 rpm for 48 h. After completion of the reaction, the cellulose tubing was removed and washed with water. Unreacted indole was extracted with ethyl acetate (2 × 50 ml) and the aqueous phase was concentrated under reduced pressure to approximately 50 ml. The halogenated tryptophans were purified using manual reverse phase column chromatography. The pure samples were then treated twice with 20 ml 0.1 M hydrochloric acid to convert the tryptophan to the hydrochloride salt, removing the solvent under reduced pressure. The salt was then dissolved in 50 – 100 ml water and lyophilized to obtain the purified L-halotryptophan as a colorless, pink, yellow, or tan powder.

## Data Availability Statement

The datasets generated for this study can be found in Data Dryad (*Pisum sativum* transcriptome) (https://datadryad.org/stash/sha re/xJfxCr7hevbW7EwC6B_tR5q_8nZapNZZ0vOK92x-wFc) and GenBank [PsYUC1-8 accession numbers, PsYUC1 (MW158556), PsYUC2 (MW158557), PsYUC3 (MW158558), PsYUC4 (MW158559), PsYUC5 (MW158560), PsYUC6 (MW158561), PsYUC7 (MW158562), and PsYUC8 (MW158563)].

## Author Contributions

KD and LC performed and designed the experiments. DG, DS, and RG synthesized all tryptophan analogs. SO’C assisted with experimental design and managed the project. KD, LC, and SO’C all wrote the manuscript. All authors contributed to the article and approved the submitted version.

## Conflict of Interest

The authors declare that the research was conducted in the absence of any commercial or financial relationships that could be construed as a potential conflict of interest.

## Footnotes

1 http://www.bioinformatics.babraham.ac.uk/projects/fastqc/
