# Supplementary material for: Nicotiana benthamiana as a Transient Expression Host to Produce Auxin Analogs
Source: Front Plant Sci. 2020 Nov 20;11:581675. doi: 10.3389/fpls.2020.581675 (PMC7714751; doi:10.3389/fpls.2020.581675)

Supplementary Material

Nicotiana benthamiana as a transient expression host to produce auxin analogues

Katharine Davis[1], Danai S. Gkotsi[2], Duncan R. M. Smith[2] Rebecca J. M. Goss[2], Lorenzo Caputi[3], Sarah E. O'Connor*[3]

[1]Department of Biochemistry, University of Cambridge, Tennis Court Road, Cambridge, CB2 1QW, UK.

[2]School of Chemistry, University of St. Andrews, North Haugh, St. Andrews, KY16 9ST, UK.

[3]Max-Planck Institute for Chemical Ecology, Department of Natural Product Biosynthesis, Hans-Knöll-Straße 8, 07745 Jena, Germany.

*** Correspondence:**
oconnor@ice.mpg.de

1 Supplementary Tables

Table S1. Sequences of the TAA and YUC genes used in this investigation. [A] sequences from TAIR (https://www.arabidopsis.org), respective accession numbers for AtTAA1, AtTAR2 and AtYUC6, [B] sequences retrieved from pea seed transcriptome. Genbank accession numbers of the *Ps*YUC sequences are the following: *Ps*YUC1 (MW158556), *Ps*YUC2 (MW158557), *Ps*YUC3 (MW158558), *Ps*YUC4 (MW158559), *Ps*YUC5 (MW158560), *Ps*YUC6 (MW158561), *Ps*YUC7 (MW158562), *Ps*YUC8 (MW158563).

Gene	Sequence
AtTAA1[A]	ATGGTGAAACTGGAGAACTCGAGGAAACCCGAAAAAATTTCGAACAAGAACATCCCC<br>ATGTCCGATTTCGTGGTCAATCTGGATCATGGTGATCCAACGGCGTACGAAGAATACT<br>GGAGGAAGATGGGTGACAGGTGTACGGTGACGATACGTGGTTGTGATCTCATGAGTTA<br>CTTCAGCGACATGACGAACTTGTGTTGGTTCCTTGAGCCAGAGCTTGAAGATGCGATC<br>AAGGACTTGCACGGTGTTGTTGGTAACGCTGCGACGGAGGATCGGTACATAGTGGTTG<br>GGACCGGTTCGACGCAGCTTTGTCAAGCCGCCGTCCACGCACTCTCTTCACTAGCCAG<br>GAGTCAACCTGTCAGCGTCGTCGCCGCCGCTCCTTTTTACTCCACATATGTGGAGGAGA<br>CGACATATGTTCGGTCGGGTATGTACAAGTGGGAAGGAGACGCATGGGGTTTCGACAA<br>AAAGGGTCCGTACATCGAGCTAGTGACGTCACCTAATAACCCTGACGGAACCATCAGA<br>GAGACGGTGGTGAACCGTCCAGACGACGACGAAGCCAAAGTGATCCATGACTTTGCTT<br>ATTACTGGCCCCACTACACTCCCATCACTCGCCGTCAAGACCATGACATCATGCTCTTC<br>ACTTTCTCCAAGATCACAGGCCACGCTGGGTCCCGTATTGGGTGGGCATTGGTGAAGG<br>ACAAGGAGGTAGCTAAGAAGATGGTTGAGTATATTATTGTGAACTCGATTGGTGTGTC<br>TAAGGAGTCACAGGTTCGAACAGCTAAGATACTCAACGTTCTAAAGGAGACTTGTAAG<br>AGCGAGTCCGAGTCTGAGAATTTCTTCAAGTATGGTCGTGAGATGATGAAGAATCGGT<br>GGGAGAAGCTACGTGAAGTTGTGAAAGAGAGCGATGCTTTCACTCTTCCCAAGTACCC<br>TGAAGCATTTTGCAACTACTTTGGAAAATCACTCGAATCTTACCCTGCGTTTGCGTGGC<br>TAGGGACGAAGGAAGAGACGGATCTGGTAAGTGAATTGAGGAGACACAAGGTAATGA<br>GCAGAGCTGGAGAGCGTTGTGGATCTGACAAGAAGCATGTCCGAGTCAGCATGCTTAG<br>TCGTGAAGACGTTTTCAATGTCTTTCTCGAGAGACTCGCCAACATGAAGCTCATTAAAA<br>GCATTGACCTTTAG
AtTAR2[A]	ATGGGACAGATTCCGAGGTTTCTTTCTTGGAGGAATATGTTGGTCCTCTCGTTGGCCAT<br>CAACTTCAGCTTGATTCTAAAGATTTTGAAGGGTGATAGAGAACGAGGAGATTCATGG<br>GACAGAACAGCGTATGTTAGCATATGGCCCGTGGTATCCACCACGGCTTCAGAATCTT<br>CTTCGTTGTCTTCAGCATCTTGCAACTATAGCAAGATTGAAGAAGACGATGATAGAAT<br>TATCAATCTCAAATTTGGTGATCCAACGGTGTATGAGAGATATTGGCAGGAAAATGGA<br>GAGGTGACAACAATGGTGATACCTGGATGGCAATCTCTTAGCTATTTTTCAGATGAAA<br>ACAACCTCTGTTGGTTTCTTGAGCCAGAGCTTGCCAAAGAGATTGTGAGGGTGCATAA<br>GGTTGTTGGGAATGCTGTAACGCAAGACCGCTTCATTGTTGTTGGCACTGGCTCAACAC<br>AATTGTATCAGGCTGCTCTCTATGCTCTCTCCCCACATGATGACTCCGGTCCCATTAAT<br>GTCGTGTCAGCCACACCCTATTATAGTACATACCCGTTGATTACAGACTGCCTCAAATC<br>AGGTTTATATCGATGGGGTGGAGATGCAAAGACGTACAAAGAAGATGGTCCATACATT<br>GAACTTGTTACATCTCCAAACAACCCTGATGGGTTCTTGAGAGAATCAGTAGTGAACA<br>GTACTGAAGGTATATTGATCCATGATTTGGCTTACTATTGGCCACAGTATACACCGATA<br>ACATCACCAGCTGATCACGATGTTATGCTCTTCACTGCTTCAAAGAGCACTGGCCATGC<br>AGGGATACGGATTGGATGGGCTTTGGTGAAAGACAGAGAGACGGCTAGGAAAATGAT<br>AGAGTACATTGAACTCAACACGATTGGGGTTTCAAAGGACTCACAGCTTAGAGTAGCC<br>AAGGTTCTTAAGGTTGTGTCAGACAGTTGTGGGAATGTAACGGGCAAATCTTTCTTTGA

	CCATAGTTATGATGCTATGTATGAGAGGTGGAAACTATTGAAACAAGCAGCAAAGGAT<br>ACTAAACGTTTCAGTGTTCCTGATTTCGTCTCTCAACGTTGCAATTTCTTTGGCAGGGTC<br>TTTGAGCCACAACCAGCATTTGCATGGTTTAAGTGTGAAGAAGGGATAGTGGATTGTG<br>AGAAGTTTCTTAGAGAGGAGAAGAAGATTCTAACTAAAAGTGGAAAGTACTTCGGAG<br>ATGAGCTAAGTAATGTGAGGATAAGCATGTTGGATAGAGATACTAACTTTAATATTTT<br>CCTTCACAGGATTACATCTTCCTTTAATTCAACTTTGTAA
PsYUC1^{B}	ATGGATCCTTTCAAACAAAAACCTAAGTCTTTATTCATTCATGGACCTATCATTGTAGG<br>TGCTGGTCCTTCTGGTATAGCAGTTGCTGCTTGTTTATCAGAACAAGGTGTTCCGAGTC<br>TTATTCTTGAGAGAAGTGACTGTATAGCTTCCCTTTGGCAAAATAGAACCTATGACCGT<br>TTGAAACTTCATTTACCTAAACATTTTTGTGAGCTTCCTTTGATGAGTTTCCTCAAGAT<br>TTTCCTATGTATCCTACCAAACATCAGTTCATTTCTTACATGGAATCCTATGCTGATCA<br>GTTTGGTATTCGTCCTAGGTTCAATCAAACTGTTGTTACTGCTGAGTTTGATCCAAGTT<br>CGGAAATTTGGAATGTTAAAACACTAGATGGTTTTCAGTATTCTTCACCTTGGCTTGTT<br>GTTGCTACCGGTGAAAATGCTGAACCTGTTATTCCTAAGATTCATGGTATGGAACATTT<br>TCATGGTCCTGTTGTTCATACTTGTGACTATAAATCTGGTTCTCAATACAAAAACAAGA<br>AGGTTTTGGTCATTGGTTGCGGTAATTCTGGTATGGAAGTTAGCTTGGATCTTTGTAGA<br>CACAATGCTCTACCTCACTTGGTTGCTAGAAACACAGTACACATGCTTCCAAGAGATA<br>TCTTTGGGTTCTCAACATTTGGAGTAGCTATGACATTAAACAAATGGCTTCCATTAAAA<br>TTAGTAGACAAGTTCCTCTTATTAGTTTCAAGCTTCTTCTTAGGAAACACCAATCACTA<br>TGGTATCAAAAGGCCTAAAACTGGTCCTATAGAGCTTAAGCTTGCAACAGGAAAAACT<br>CCTGTACTTGATGTTGGTCAAATTGCTCAAATTAAATCCGGTAACATTAAGGTGATGGA<br>AGGTGTGAAGGAGATAACAAGGAATGGTGCAAAATTTATGGATGGACAAGAAAAGGA<br>ATTTGAAGCAATAATCTTAGCAACAGGTTACAAGAGCAATGTGCCTAGTTGGCTTAAG<br>GGAAGTGATTTTTTCACTAAAGATGGAATGCCGAAAACACCCTTTCCTCATGGATGGA<br>AAGGAGAGCAAGGATTGTATACGGTAGGGTTCACGAGAAGAGGTCTACATGGAACAT<br>ATTTTGATGCTATCAAAATATCTGAAGATATTACAAGTCAATGGAAAACAGTTAAGAG<br>CAAGAGTTGTAGTGATTCACATATCATAAACCTTATTAGTACTACTACTCTTAATAATG<br>TTTAG
PsYUC2^{B}	ATGGAGAACTTGTTTCGCCTAGTTGATAACCAACAAGACTCAATCTCACCTCGTTGCAT<br>TTGGGTTAACGGTCCTATAATCGTAGGAGCAGGACCATCAGGTTTAGCAGCAGCGGCA<br>TGTCTCAAACAAAAAAACATTCCATCCATAATTCTCGAAAGATCCAACTGCATAGCTT<br>CATTATGGCAACTCAAAACCTACGATCGGTTACGTCTCCACCTGCCAAAACAATTCTGC<br>GAACTACCCTTCATGGAATTTCCATCGAATTTCCCAACATACCCTTCAAAGCAACAGTT<br>CATTAAGTACCTGGAGAATTACGCGGAAACGTTCCATATTCGTCCGAAATTCAATGAG<br>ACAGTGAAAAATGCCGAATTCGATAGTAAAATCGGTTGCTGGAAGTTGAAGTGTCAAA<br>GTAGTTTTAAAGGCGATGTTACGACGGAGTATGTTTGTCGGTGGTTGATCGTCGCGACC<br>GGAGAGAATGCCGAGGCTGTTGTGCCTGATATTGAAGGTGCAGATGAGTTTGAAGGAG<br>TTATAAGACATACAAGTTTGTATAAAAGTGGTGAAGAGTTTAGAGGGAAGAGAGTTTT<br>GGTTGTTGGGTGTGGAAATTCTGGTATGGAAGTTTGTTTGGATCTTTGTAACCATGATG<br>CTACTCCTTCTCTGGTCGTTAGAGATTCAGTACACGTTCTACCACGAGAGATGCTAGGA<br>AAATCAACTTTTGGGTTGTCCATGTGGTTACTAAAGTGGTTTCCATTGAGACTTGTCGA<br>TCGTTTCTTGCTGTTAGTGTCATGGCTTTTGCTTGGTGACACTTCTCAACTTGGTTTGGA<br>TCGTCCTACTTTGGGTCCCCTTCAACTCAAAAACCTCACGGGAAAAACTCCTGTCCTAG<br>ATGTGGGTACCCTTGCCAAGATTAAAGCTGGACACATTAAGGTAAGGCCAAGCATAAA<br>GAAGTTAAAACGTCATACAGTGGAATTTGTAGATGGAAGATCAGAGAATTTTGATGGC<br>ATCATATTGGCAACTGGTTACAAAAGCAATGTACCCTATTGGCTCAAGGAAGAGGATA<br>TGTTTTCTATGAAAGATGGATTCCCTATGAAGCCATTTCCCAATGGATGGAAAGGTAA<br>AAATGGACTCTATGCAATAGGTTTTACAAAAAGAGGACTATTAGGTGCATCAATGGAT<br>GCAAAAAGAATAGCTAATGACATTGAACAATGTTTAAAAGCTGAGGCAAAACATGGA<br>TCA
PsYUC3^{B}	ATGCTCATGAACTACTTAAAGGAACTAGAATGCAAAATGGTACATGATCATCAAAAG<br>AAGGACAAAATGGTAACTTCAATATTGGTTCAAGGACCAATTATAGTAGGAGCTGGTC<br>CATCAGGATTAGCAGCAGCAGCATGTCTTAAACAAAAAGGAATTCAAAGCTTAATCCT<br>TGAAAGAGCAAATTGTTTAGCTTCAATGTGGCAACTCAAAACCTATGATAGATTAAAG

	CTTCATCTTCCAAAACAATTTTGTCAATTACCTCTTATGCCATTTCCAAAAGGGTTACC ATCATATCCAACAAAGCAACAATTTTTATCTTACTTAAAAGCCTATGCTAATCACTTTG ATATTAATCCTATTTTTGGTAAACAAGTTGTGAATGCTGAATTTGATCTTACTTGTGGA GTTTGGAGAGTGAAGACTCAAGAAATTATTATGAAAAAGTGTATTGTTATTGAGTATG TTTGTCAATGGTTGATTGTTGCTAGTGGTGAAAATGCTGAGGAAGTTATGCCATCAATT GAAGGGATGGAACAATTTCAAGGACCTATTTTGCATACTAGTTTGTATAAAAGTGGAA GCATGTTTTGTGGGAAGAATGTTTTGGTGGTGGGGTGTGGAAATTCAGGCATGGAGGT GTGTTTAGATCTTTGCAACCATAATGCTCATCCTTCCTTAGTTGTTAGAGATACGGTAC ATATCTTGCCACAACAAATATTTGGAAAATCAACTTTTGGTTTATCTATGTGGTTACTT AAATGGTTCTCAGTACATTTTGTGGATCAATTTTTACTTCTAATGTCATATTTCATACTT GGTGACACATCTCAATATGGAATTCAAAGGCCAAAAATTGGTCCTTTAGAGCTTAAGA ATTTGTATGGAAAAACACCAGTTTTAGATGTTGGGACAGTAGCTCAAATCAAAACTGG CAAAATTAAGGTCTGCAAAGGAATTAAACGACTAGCACATAATGCAGTGGAGTTTGTT GATGGAAAAGTAGAGAACTTTGATGCAATCATTTTAGCTACTGGTTACAAAAGCAATG TGCCCTCTTGGTTAAAGGGAAGTGACATGTTTAGTGAGAAAGATGGTTTCCAAGGAA ACCATTCCCAAATGGATGGAAAGGTGAAAAAGGATTATATGCTGTTGGTTTCACCAAA CGTGGCTTACTTGGTTCATCTATTGATGCAAAGAGAATTGCTGAAGATATTGAACATA GCTGGAAAGCTCTTAAGGCCAAGCCACTTGCA
PsYUC4[B]	ATGAAAAACAAAACCATGGAAATCCAAATGCCAGTTGTGATTGTAGGTGCTGGGCCTG CTGGGTTAGCAACCTCTGCATGTCTCAACAAACTTTCAATCCAAAACATTGTACTAGAA AGAGATGACTGTCATTCCTCTCTTTGGAGGAAAAGAACCTATGATCGTTTGAAACTTCA CTTAGGTAAAGGCTTTTGTAACCTACCTCACATGCCATTTTCCTCTGATCTTCCAGTGTT TATCCCTAGGGTTGATTTCCTTCGGTATTTGGATGATTATGTGAGTACTTTTAAGATCTT CATCCGCTACAACCGGTATGTCCACGAAGCTTCTTTCGATGTCAAGACCGGAAAATGG AGGGTTTGTGTGATGGATAGTGCTCTGAATGTTGGTGAAGTTTATGTTGCTGATTATTT AGTGGTTGCTTCCGGAGAGAGCTGTGATGCTTATATTCCGAAGATAACCGGGCTTGAT AAGTTTGAAGGTGAATTCTTTCATTGCACCAAGTATCAGAATGGAAGGCCTTTTTATGA TAAGAATGTGTTGGTTGTTGGTAGTGGAAATTCTGGTATGGAGATTGGTTATGATCTCT CTACTTGGGGTGCAAATACCTCCATGGTTATAAGAAGTCCGGTACATTTTTTAATAAAA GAAATGGTGTACATTGGAATGTCTTTGCTGAAATATGTAAGTGTTGAAAATGTGGACA AGCTTATGTTGGTTATGAGCAAATTGTTGTATGGAGATTTGTCTAAGTATGGCTTGATT AGGCCAAAGGAAGGACCCTTTGCATTGAAATTGAAGGGTGGTCGGACTCCTACAGTTG ATGTCGGTACCATCAAACACATCAAAGAAGGAAAAATAAAGGTACACTCTGAAATTTC AAGCATAAAAAACGGCAAGACCATTGAATTTGTAGATGGAAAAACTGGTCAATTTGAC GTCATAATCTTTGCTACGGGATACAGAACCAATGTGCACAAGTGGCTTAAGGATTACA AAGATTTGTTTAATGAAAATGGAATGCCAAAACCTGCTTATCCAAATCACTGGAAAGG AGAGAATGGAATCTACTGTGTTGGATTCTCAAAAAGGGGATTACAAGGCATTAACTAT GATGCTCAGAAAGTAGCAAGGGATATCAGTGTCACTATCAATGCAAGGAAGAAGATA CTTACAGCTGATGAGGCCAATGATGCTCAAATCAAGCTATTAGAT
PsYUC5[B]	ATGGAGAATTTGTTTCGTCTTGCTGATCACGAAGATTTTATCTCACGCCGTTGCATTTG GGTCAATGGACCTGTGATCATTGGCGCAGGTCCATCTGGCCTAGCAACGGCGGCATGT CTTAGAGAACAAGGAGTACCATTCCTTGTTGTCGAAAGAGCTGATTGCATAGCATCAC TTTGGCAAAGAAGAACCTATGACAGGTTGAAACTTAACCTCCCAAAACAATTCTGTCA ACTCCCTAACCTTCCATTCCCTGATGATTTCCCTGAATACCCTTCAAAGAAACAGTTCA TAAACTACCTTGAAACCTATGCTAACAAATTTGAAATCAAGCCGCAATTCAACCAGTG TGTTCAATCTGCTAAGTATGATGAAACCAGTGGATTATGGAGGGTGAAAACCAATGAA GTTGAGTATATTTGCAGGTGGCTTGTTGTTGCTACCGGCGAAAATGCTGAGTGTGTTAT TCCTGAAATTGAAGGACTTTCTGAGTTCAAAGGTAAAGTTGTTCATGCTTGTGATTACA AGTCAGGCGAAAATTTCAAAGGAAAGAAAGTTCTCGTTGTTGGTTGTGGAAATTCAGG ATTGGACATCTCACTTGATCTTAGCAACCACCATGCTTTACCTTCTATGGTTGTTCGTA GCTCGGTTCATGTGTTACCTTCAATGGGTCCTTTGCAGCTGAAGAACACAGAAGGAAA AATACCTGTTTTGAATATTGGTACCTTGGAGAAAATTAGATCTGGAGATATAAAAGTT GTCCCTGGAATCAAGAAGTTTAACAAAAACGGCGAAGTCCAGCTTGTTAATGGCGAAA

	AGATTGATGTTGATGCAGTTGTTCTTGCTACCGGTTATAGGAGCAATGTTCCTTCTTGG<br>CTTCAGGAAGGTGAATTTTTCTCGAAAAACGGATACCCGAAGATGCCATTTCCACATG<br>GATGGAAAGGAAATTCAGGACTATATGCTGTAGGGTTCACAAAGATAGGGCTTTTTGG<br>TGCTTCATATGATGCTGTTAAAATTGCACAAGATATTGGAAATGTTTGGAAACATGAG<br>ACTAAACAGAAGAAACAACGCTTGTCA
PsYUC6^{B}	ATGCAGGTAGCACCGGTGATAATAATCGGAGCCGGAACTTCCGGCATAGCCACGGCA<br>GCTTCTTTAACAAAACAATCCATACCATTCATCATTCTCGAAAGAGAAAACTGTTTTGC<br>TTCTCTATGGCAAAACTACACCTATGATCGTCTTCATCTTCATCTAAGAAAGCAAATTT<br>GCGAGTTACCATATTTTCCATTTCCATCTTCTTATCCTAACTACGTACCAAAAAAGCAG<br>TTCATACAGTATCTAGACAGCTATGTTAAAAACTTCAACATCGATCCTTTATACAACAG<br>AAAGGTTGTGTTAGCGGAGTATTTTGAGGATGATGAGAAATGGAGGGTTGAGGCTGAG<br>AATAGAAGTTCCGGTGAAGTTGAAGAATATTCTGGAAGGTTCTTGGTGGTGGCTAGTG<br>GCGAAACGGCTGAGCCTCGTGTACCGGAAGTTTTAGGGTTGGAGAGTTTCAATGGGAA<br>AGTGATGCATTCGACTGGGTATAAAAATGGGAGAGAGTTTAAAGATAAACATGTTCTT<br>GTTGTTGGTTCTGGGAATTCTGGTATGGAGATTTCTTTAGATTTGGCTAATTTTGGTGCC<br>AAACCTTCTATCATTGTTAGAAGTCCGGTTCATTTTCTTTCAAGGGATATGATGTATTA<br>TGCTGGTGTTTTGATGAGTTATCTGTCACTAAGCACAGTGGAGAAACTAGTTGTGATAG<br>TTAGCAGAATTGTGTATGGAGATCTGAGTAAATATGGTATACCTTTCCCTAGTGAGGGT<br>CCTTTCACTATGAAGAATAAGTATGGAAAATTTCCTATAATTGATTTGGGAACGGTTAA<br>GAAAATCAAATCTGGAGAGATACAGGTGTTGGGAGCTGAAATAGAGAGTATAAGAGG<br>TAATCAAGTGTTGTTCCGTGATGGAAAATCCTACCCATTTGACTCCATTATATTCTGTA<br>CAGGCTTCAGCAGATCAACTCAAAAATGGCTTAAGGGAGGTGATGATCTTTTGAATGA<br>GGATGGTTTTCCAAAGCCTGGTTTACCATACAATTGGAAGGGTAAGAATGGTTTGTAC<br>TGCGTTGGATTGTCAAGGAGGGGGTTTTATGGAGCTAACCTTGATGCTCAAAATGTAG<br>CAAATGATATTGCCTTGTTAGTTCCTCAAGACGAAAGAAAA
PsYUC7^{B}	ATGCAAGAATTCACAGTTGTAATTGTTGGTGGTGGTCCTTCTGGCCTAGCAATTTCAGC<br>TTTACTAATACAAAACTCAATCTCTCACACTATACTCGAAAAAGAAGATTGTAATGCTT<br>CTCTTTGGAGGAAAAATGCTTACGATCGTCTAAACCTCCATTTAGCTAGTGAGTTTTGC<br>TCTTTACCTCTCGTGCCGCATCCACCTTCAGGCCCAACATACCTAACCAAAGATCAATT<br>TCTCCAATACATAGATGAATATGTCGATCATTTTGACATAAAACCTCGTTATTATCGTG<br>TCGTTGAGTCCGCTAACTACGATAATGTTCAAAACAAATGGGTTGTTGAAGCCAAAAA<br>CACCCTAGAAGGTACCTTGGAAGTTTATGGGGCAAAGTTTCTAGTCATTGCCTCTGGCG<br>AAAATAGTGAAGGTTTTATTCCCAATGTTCATGGATTAGGGAAATTTGAAGGAGAGGT<br>GGTACACTCCAAGTACTACAAATCTGGTTCAAAATACAAATCAAAAGATGTTTTGGTT<br>GTTGGCTGTGGTAACTCAGGAATGGAGATTGCATATGATCTCCATAACTGGGGTGCTA<br>ACACTTCCATTGTCATTCGAAATCCGCTTCATGTCTTCACCAGAGATATGATTCGTATA<br>GGGATGCGTTTGGTGCAATATTTTCCTGTTTATATTGCTGATACAATCATTACAATTCT<br>AGCAAAGTTAAAATATGGTGATCTGTCAAAATACGGGATTCATCGTCCTAAAGATGGA<br>CCTTTATATCTCAAAAACAGTACAGGAAAATCTGCAGTTATTGACGTAGGAACCATTG<br>AAAAGATTATGGAAGGATCCATAAAGGTCGTTCCTTCAGGTATCAAGAAAATCGAGAA<br>GAAGAATGTTATCTTTGAAAACAATATGGAGAAAGAGTTTGATGCCATTGTTTTTGCTA<br>CCGGCTACAAAAGCGTAGCTAATGAATGGCTAAAGGATTACAAATATGCGCTTAATGA<br>GAAAGGGTTCCCTAAAAATCCTTTTCCGAAACATTGGAAGGGAGATCATGGATTGTAC<br>TGTGCAGGACTAGCAAGGAAAGGTTTGTTTGGAGTCAAAAAGGATGCTGAGGCAATTG<br>CAGAAGACATCAACCGAACTCTTAAGTTGGAGAAT
PsYUC8^{B}	ATGAAGAAAGAGAATCAAACAGTGATCATTGTTGGAGCAGGCCCTTCTGGTCTCTCTG<br>TAGCAGCATCCTTAACAAACCAATCCATTCCTTACATAATCCTCGAAAGAGAAAACTG<br>TTTTGCGTCTCTATGGAAAAAATATGCATACGACCGTCTTCATCTTCACCTCAAAAAGC<br>AATTTTGTGAGCTTCCACATAAGCCATTTCCACCTTCCTTTCCATCATACATTCCTAAGG<br>AAAAGTTTTTGCAGTATTTAGAAGACTATGTATCTCACTTCAAGATCAATCCTGTGTAT<br>CAGAGGACAGTGGAGTATGCGGAGTTTGATGAAGGCTGTGAGAAATGGAAGGTGAAG<br>GCTGTGAATAAGGATTCCGGCGAAGTTGAGGAATACGATGGAAGGTTTTTGGTTGTGG

	CTAGTGGTGAAACTAGTGACCCTTTTGTACCTGAGATTGAAGGGTTGAAGAGTTTTGGT<br>GGGAAGGTGATTCATTCAACCAGGTTTAAGAATGGGAAAGAGTTTAAAGATGAACAT<br>GTTCTTGTTGTTGGGTCTGGGAATTCTGGTATGGAAATTGCATTGGATCTTATCAACCA<br>TGGTGCTAAAACTTCAATCCTCGTTCGAAGCCCGGTTCATATATTGTCAAAAGGAATGC<br>TGAATTTAGGCTTATTTATGATGAAGTATTTGTCAATGAAGATAGTGGATTCATTAATG<br>GTGATGCTTAGTAAACTGGTTTATGGTGATATGACTAATTATGGAGTTGGTAGGCCTAA<br>TGAGGGACCTTTTTACATGAAAGTTAAGTACGGCAAGTATCCAGTTGTTAATGTTGGA<br>ACCTATCAGAAAATTAAATCTAAAGAGTTAAAGGTGTTGCCAACGGAGATAGAATGCT<br>TAAGTGGCAATAATGTGTTGTTCAAGAATGGTGAATTGCATACTTTTGACTCTATTATT<br>TTCTGCACTGGTTTCAAGAGATCGACACATAAGTGGCTTAAGGGAGATGATTATCTTTT<br>GAGTGATGATGGTATTCCGAAGCAAAGTTACCCTTTTCATTGGAAGGGAAAGAATGGT<br>TTGTATTGTGTTGGATTATCAAGGAGAGGCTTATATGGAGCTGCTCAAGATGCTGAAA<br>ATGTAGCAAATGATGTTAGATCCATCATGCAAGAAATCATA
AtYUC6[A]	ATGGATTTCTGTTGGAAGAGAGAGATGGAAGGTAAACTAGCACATGACCACCGCGGC<br>ATGACGTCACCGCGTCGTATCTGCGTCGTCACCGGTCCGGTGATCGTAGGCGCCGGAC<br>CGTCGGGACTAGCCACGGCAGCATGTTTAAAAGAGAGAGGTATCACGTCCGTACTACT<br>AGAGAGATCAAACTGTATAGCATCACTATGGCAGCTCAAGACTTATGACCGTCTTCAT<br>CTTCACCTTCCTAAACAATTCTGTGAACTTCCGATTATACCCTTCCCCGGAGATTTCCCT<br>ACCTACCCGACGAAGCAACAGTTCATCGAGTACCTTGAGGACTACGCTCGGAGGTTTG<br>ACATAAAGCCGGAGTTTAACCAAACGGTTGAGTCGGCTGCGTTTGATGAAAACCTTGG<br>GATGTGGCGCGTGACTAGCGTGGGAGAAGAAGGCACGACGGAGTATGTTTGTCGGTG<br>GTTAGTGGCGGCGACGGGGGAGAATGCGGAGCCGGTGGTACCTAGGTTTGAGGGGAT<br>GGATAAGTTTGCAGCCGCCGGGGTAGTTAAGCACACGTGTCATTATAAAACCGGTGGA<br>GATTTCGCCGGAAAAAGGGTTCTTGTCGTCGGATGTGGAAACTCCGGTATGGAGGTTT<br>GTTTGGATCTCTGCAACTTCGGTGCTCAGCCTTCTCTCGTTGTCAGAGACGCTGTGCAC<br>GTCCTACCACGAGAGATGTTGGGTACTTCAACTTTTGGGCTGTCCATGTTCTTACTGAA<br>ATGGCTGCCCATCCGGCTTGTTGACCGTTTCCTTTTGGTTGTTTCCCGGTTCATCCTCGG<br>GGATACTACCCTTTTAGGTCTTAACAGGCCCCGGTTAGGTCCACTCGAGCTCAAAAAT<br>ATCTCCGGTAAAACTCCGGTTCTCGACGTTGGCACGCTAGCCAAAATCAAAACCGGAG<br>ACATTAAGGTGTGTTCGGGGATAAGAAGGTTAAAACGACATGAAGTTGAGTTCGATAA<br>CGGAAAAACAGAGAGATTTGACGCCATTATATTAGCAACTGGCTACAAAAGCAACGT<br>ACCCTCTTGGCTAAAGGAGAATAAAATGTTTAGTAAGAAAGATGGATTTCCAATACAA<br>GAGTTCCCTGAGGGATGGAGAGGGGAATGTGGGCTATACGCGGTCGGATTCACAAAA<br>CGTGGGATTAGTGGAGCATCAATGGATGCAAAGAGAATAGCTGAAGACATACACAAG<br>TGTTGGAAACAAGACGAGCAACTGCAATGCAAATTGGGGAAAAGAATGAAAAGGAAA<br>TTTAGTGAGAGTGATTGTGGTGGGAATTGA

Table S2. Sequences of forward and reverse primers for each of the TAR and YUC genes featured in this report. Underlined sequences are the cloning overhangs.

Gene	Forward primer	Reverse primer
AtTAA1	AAGTTCTGTTTCAGGGCCCGGTGAAACTGGAGAACTCG AGGA	ATGGTCTAGAAAGCTTTAAAGGTCAATGCTTTTAATGAGCTTCA
AtTAR2	AAGTTCTGTTTCAGGGCCCGGGACAGATTCCGAGGTTT CTTTCT	ATGGTCTAGAAAGCTTTACAAAGTTGAATTAAAGGAAGATGTAATC
PsYUC1	AAGTTCTGTTTCAGGGCCCGGATCCTTTCAAACAAAAACCT	ATGGTCTAGAAAGCTTTAAACATTATTAAGAGTAGTAGTCTAATAAGG
PsYUC2	AAGTTCTGTTTCAGGGCCCGGAGAACTTGTTTCGCCTAGTTG	ATGGTCTAGAAAGCTTTATGATCCATGTTTTGCCTCAGCTTTTAAAC
PsYUC3	AAGTTCTGTTTCAGGGCCCGCTCATGAACTACTTAAAGGAACTAG	ATGGTCTAGAAAGCTTTATGCAAGTGGCTTGGC
PsYUC4	AAGTTCTGTTTCAGGGCCCGAAAAACAAAACCATGGAAATC	ATGGTCTAGAAAGCTTTAATCTAATAGCTTGATTTGAGCA
PsYUC5	AAGTTCTGTTTCAGGGCCCGGAGAATTTGTTTCGTCTTGC	ATGGTCTAGAAAGCTTTATGACAAGCGTTGTTTCTTCT
PsYUC6	AAGTTCTGTTTCAGGGCCCGCAGGTAGCACCGGTGATA	ATGGTCTAGAAAGCTTTATTTTCTTTCGTCTTGAGGAAC
PsYUC7	AAGTTCTGTTTCAGGGCCCGCAAGAATTCACAGTTGTAATTGT	ATGGTCTAGAAAGCTTTAATTCTCCAACTTAAGAGTTCG
PsYUC8	AAGTTCTGTTTCAGGGCCCGAAGAAAGAGAATCAAACAGTGAT	ATGGTCTAGAAAGCTTTATATGATTTCTTGCATGATGGA
AtYUC6	AAGTTCTGTTTCAGGGCCCGGATTTCTGTTGGAAGAGAGAGATGG	ATGGTCTAGAAAGCTTTAGATTTTTTTACTTGCTCGTCTTGTTTC

Table S3. MRM transitions, collision energy (CE) and retention times of compounds identified in metabolite extractions during this investigation.

Compound	MRM transition	CE (eV)	Retention time (min)
IAA	176.1 > 130.1	18	3.61
4-Cl-Trp	239.2 > 222.2	12	2.70
5-Cl-Trp	239.2 > 222.2	12	2.73
6-Cl-Trp	239.2 > 222.2	12	2.77
7-Cl-Trp	239.2 > 222.2	12	2.69
4-Cl-IAA	210.1 > 164.1	18	4.21
5-Cl-IAA	210.1 > 164.1	18	4.55
6-Cl-IAA	210.1 > 164.1	18	4.59
7-Cl-IAA	210.1 > 164.1	18	4.50
1-CH_3-Trp	190.1 > 144.1	18	2.58
5-CH_3-Trp	190.1 > 144.1	18	2.48
6-CH_3-Trp	190.1 > 144.1	18	2.48
5-CH_3O-Trp	235.2 > 218.2	10	2.05
5-CH_3O-IAA	190.0 > 158.0	10	4.82
5-OH-Trp	221.2 > 204.2	10	1.30
5-Br-Trp	283.0 > 266.0	18	2.82
6-Br-Trp	283.0 > 266.0	18	2.86

7-Br-Trp	283.0 > 266.0	18	2.75
5-Br-IAA	255.0 > 209.0	18	4.70
6-Br-IAA	255.0 > 209.0	18	4.74
7-Br-IAA	255.0 > 209.0	18	4.62

2 Supplementary Figures

Figure S1. UPLC chromatograms in MRM mode of free IAA in *N. benthamiana* leaves following infiltration with *At*TARs and *Ps*YUC7.

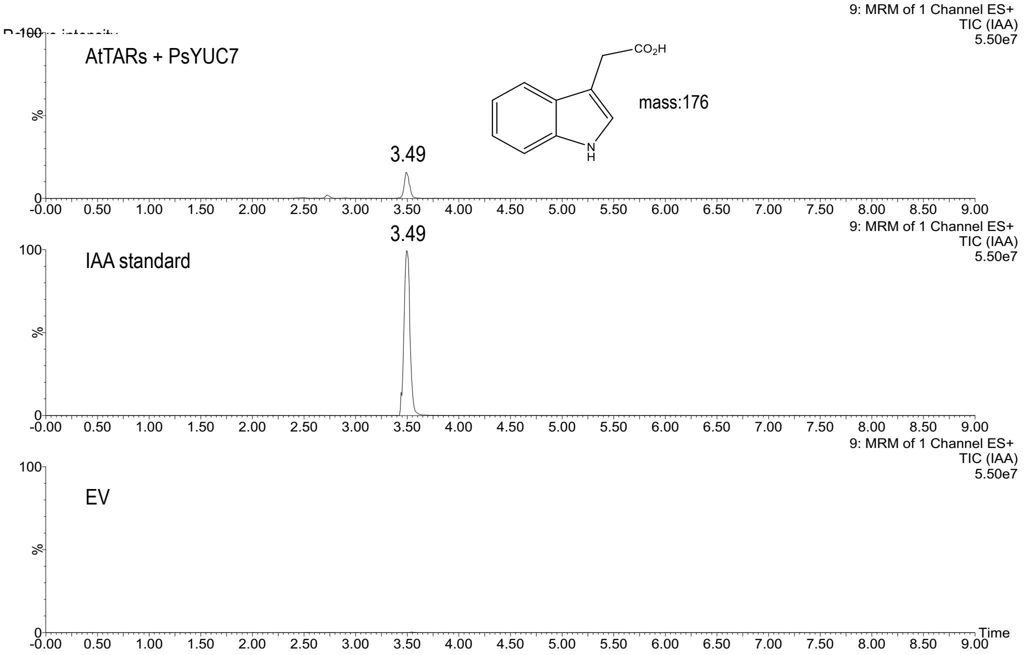

Figure S2. *N. benthamiana* plant following Agrobacterium-mediated infiltration with *At*TAA1, *At*TAR2 and PsYUC7. The transformed leaf is exhibiting a curled phenotype, characteristic of auxin over-production.

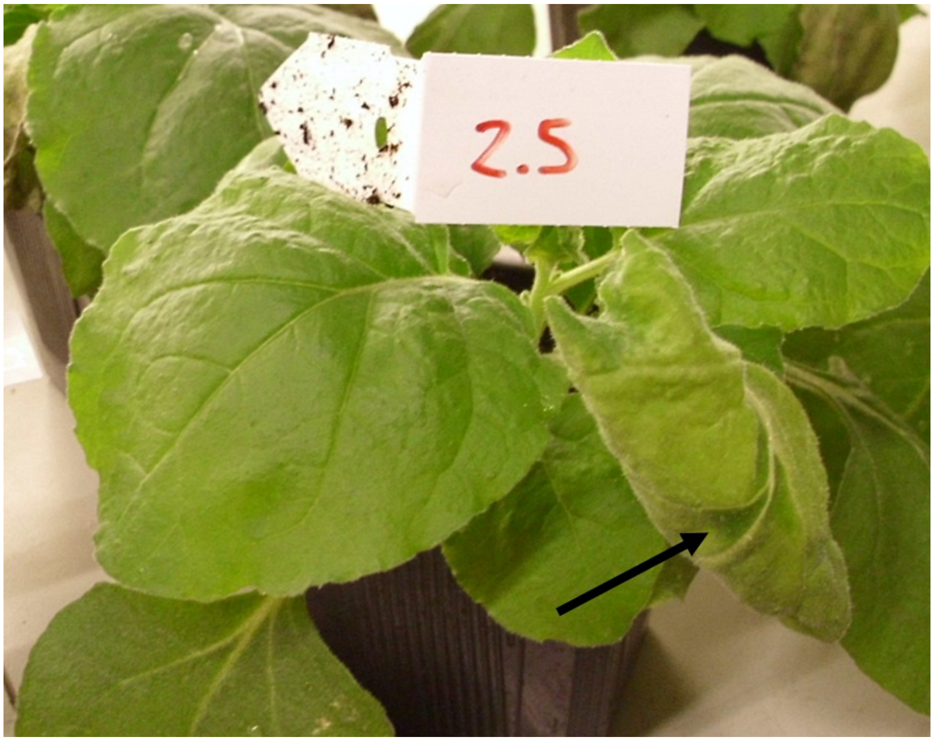

Figure S3. UPLC chromatograms in MRM mode of Cl-trp production in *N. benthamiana* following infiltration with the bacterial halogenase, A) RebH, B) PrnA, C) ThdH, D) PyrH with the partner reductase, RebF. In each case, upper panel: MRM trace of Cl-IAA from the harvested leaf extract, middle panel: MRM trace of the relevant Cl-IAA standard for each halogenase, bottom panel: empty vector (EV) negative control.

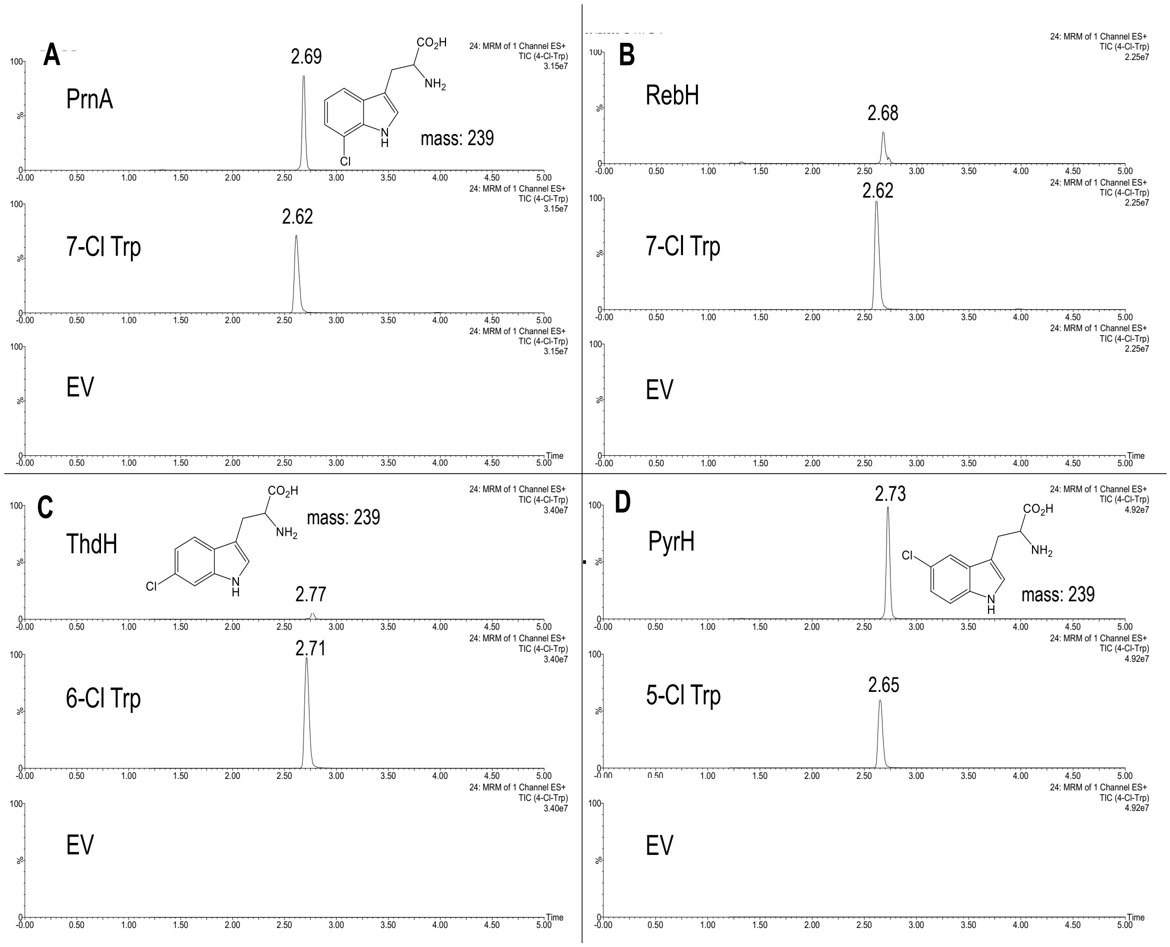

Figure S4. UPLC chromatograms in MRM mode of Cl-IAA from *N. benthamiana* leaves infiltrated with *At*TARs, *Ps*YUC7 and one of four bacterial halogenase genes; A) RebH, B) PrnA, C) ThdH, D) PyrH with the partner reductase, RebF. In each case, upper panel: MRM trace of Cl-IAA from the harvested leaf extract, middle panel: MRM trace of the relevant Cl-IAA standard for each halogenase, bottom panel: empty vector (EV) negative control.

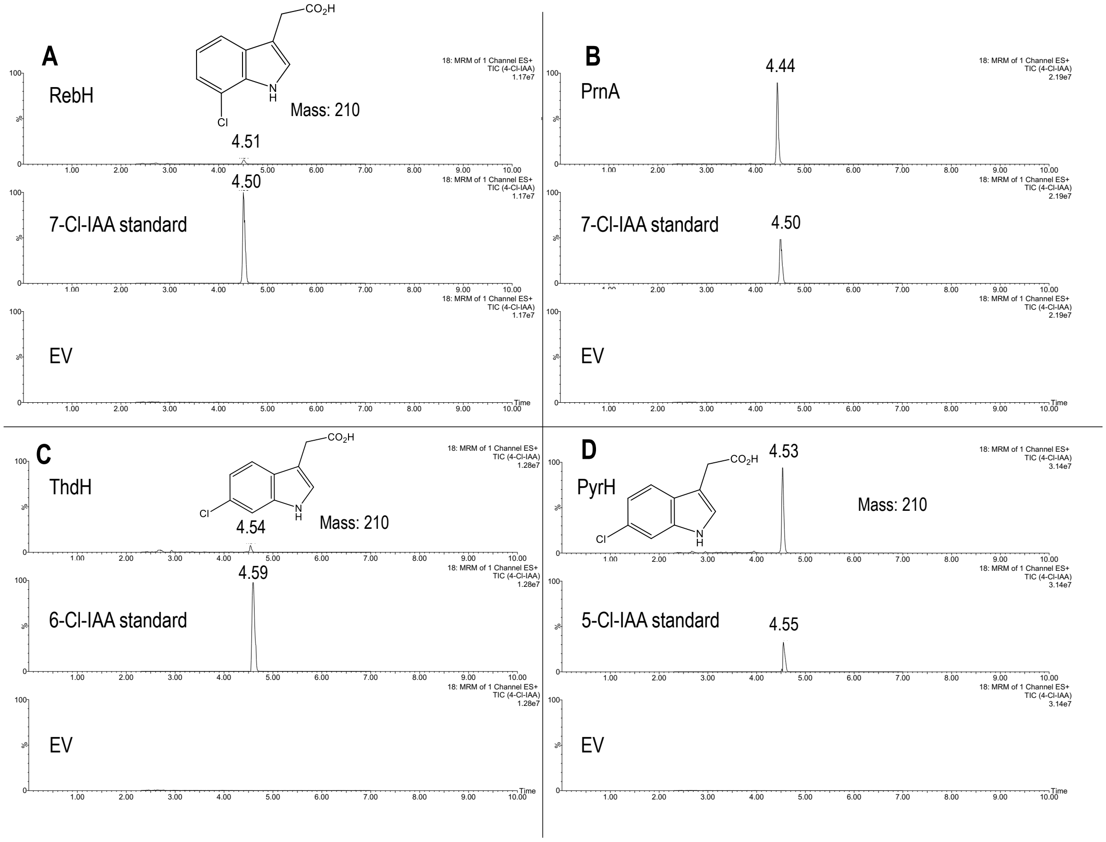

Figure S5. UPLC chromatograms in MRM mode of Br-IAA production following infiltration of *At*TARs, *Ps*YUC7 and a halogenase gene, A) PrnA, B) PyrH, with RebF in KBr buffer. In each case, the top panel: MRM trace of Br-IAA from leaf tissue. Middle panel: trace of Br trp from leaf tissue. Bottom panel: MRM trace of a 7-Br-trp standard.

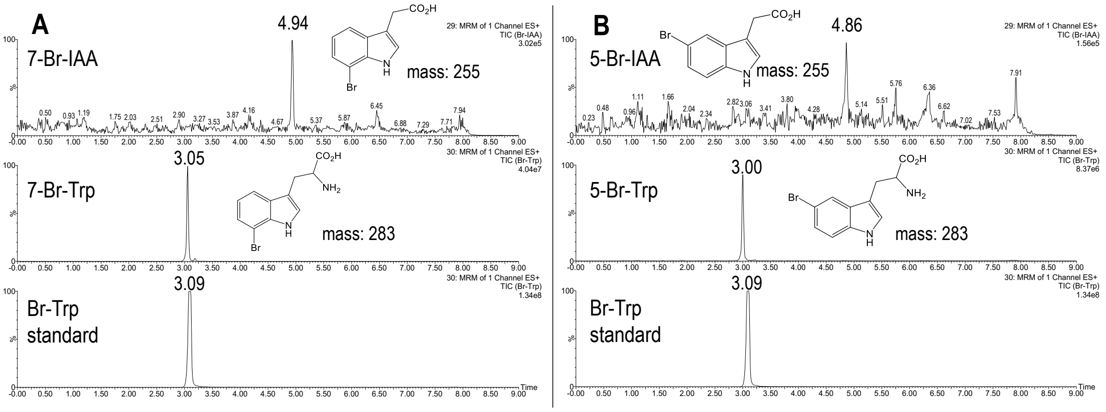

Figure S6. UPLC chromatograms in MRM mode of X-IAA production by *N. benthamiana* leaves following infiltration with X-trp. In each case A, B, C, D, the first panel: MRM trace of the X-IAA product, second panel: MRM trace of the infiltrated substrate. For C and D, third panel: MRM trace of the X-IAA product standard. 6-Br-IAA and 6-CH_3-IAA standards were unavailable.

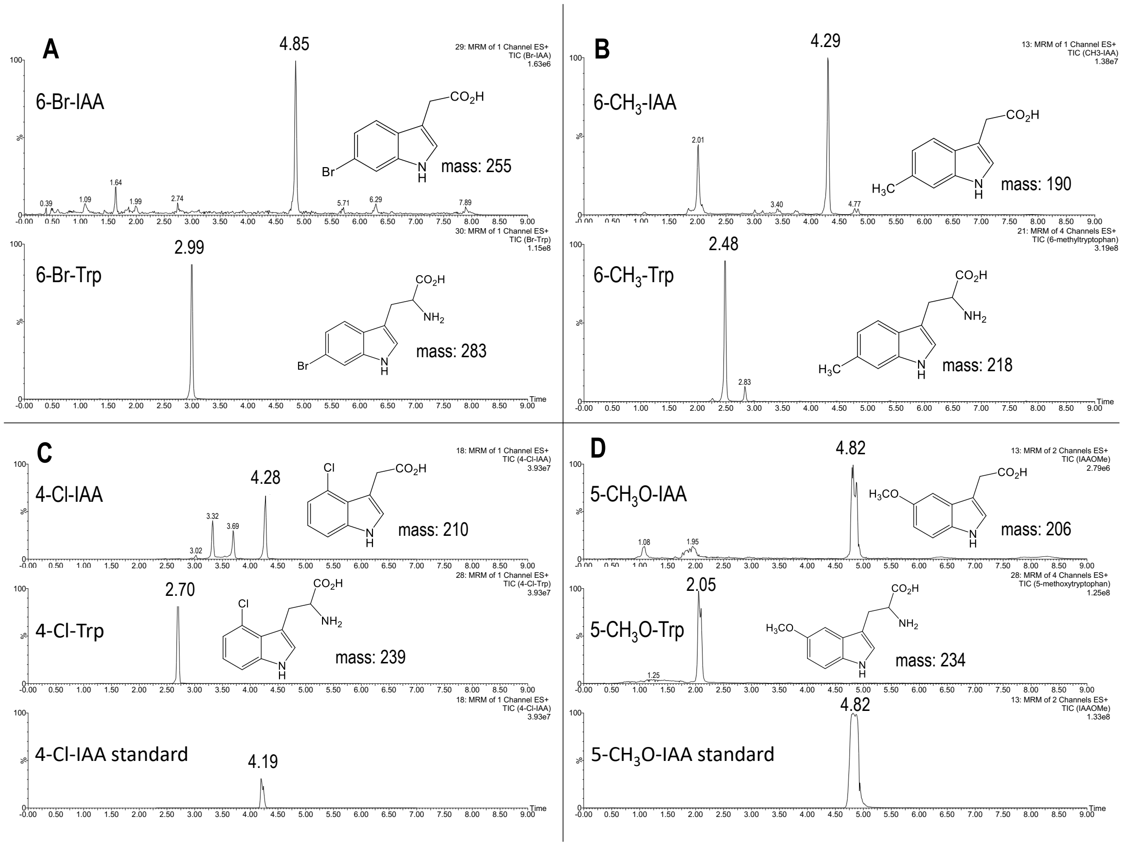

Figure S7. Cl-IAA production in *N. benthamiana* leaves from infiltration of *At*TARs, *Ps*YUC7 and bacterial halogenases in the presence of RebF, in KBr buffer. Bars represent mean ± SEM, n = 4.

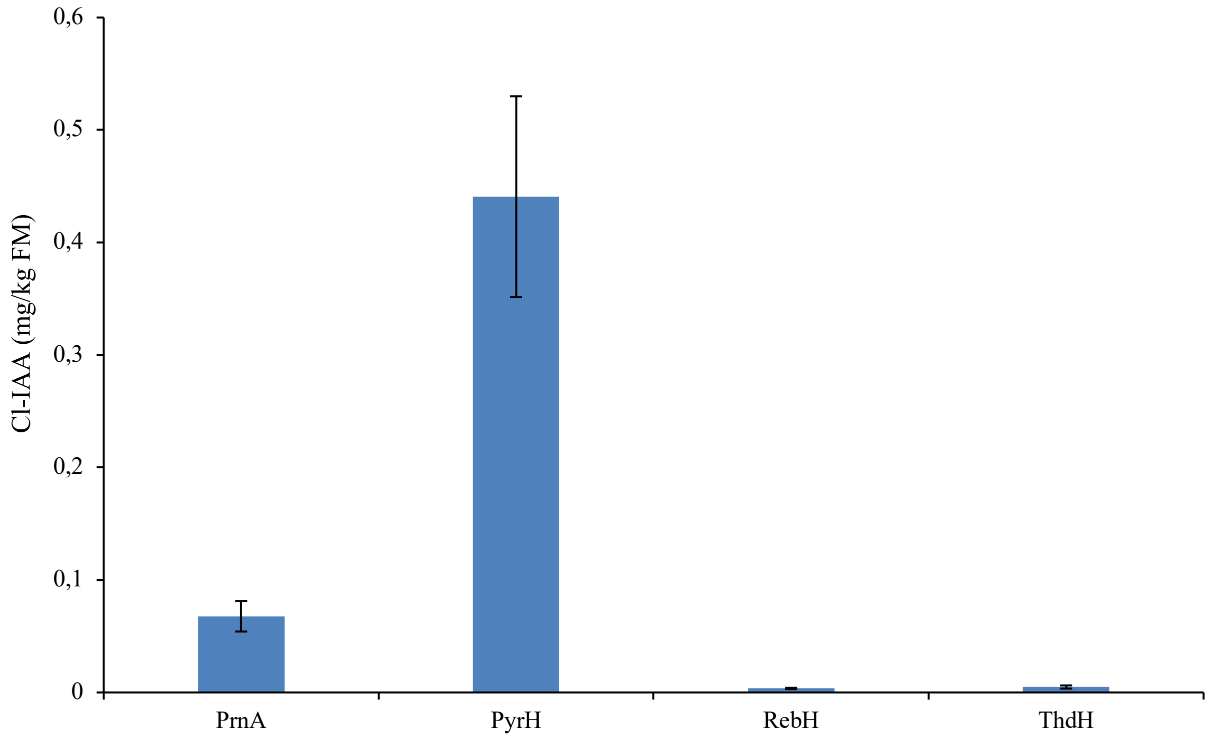

Supplement: Supplementary file 1 [file Data_Sheet_1.pdf]
